# Who Is Exposed to E-Cigarette Advertising and Where? Differences between Adolescents, Young Adults and Older Adults

**DOI:** 10.3390/ijerph16142533

**Published:** 2019-07-16

**Authors:** Kimberly G. Wagoner, David M. Reboussin, Jessica L. King, Elizabeth Orlan, Jennifer Cornacchione Ross, Erin L. Sutfin

**Affiliations:** 1 Wake Forest School of Medicine, Department of Social Sciences and Health Policy, Medical Center Blvd, Winston-Salem, NC 27157, USA; 2 Wake Forest School of Medicine, Department of Biostatistics and Data Science, Medical Center Blvd, Winston-Salem, NC 27157, USA; 3 Department of Health Behavior, Gillings School of Global Public Health, University of North Carolina at Chapel Hill, Chapel Hill, NC 27514, USA

**Keywords:** e-cigarette advertising, adolescents, young adults, adults

## Abstract

Little is known about differences between adolescents’ and adults’ exposure to e-cigarette advertising in various media channels, such as retail establishments, print, television, radio, and digital marketing. We examined the exposure to e-cigarette advertising in these channels amongst adolescents (13–17), young adults (18–25), and older adults (26+). Adolescents (*N* = 1124), young adults (*N* = 809), and adults (*N* = 4186) were recruited through two nationally representative phone surveys from 2014–2015. Lifetime e-cigarette advertising exposure was prevalent (84.5%). Overall, older adult males and older adult cigarette smokers reported the highest exposure to e-cigarette advertising (*p* < 0.001). Television was the largest source of exposure for all age groups. Adolescents and young adults had higher odds than older adults of exposure through television and digital marketing. However, adolescents had lower odds than young adults and older adults of exposure through retailers and print media. Although e-cigarette advertising appears to be reaching the intended audience of adult smokers, vulnerable populations are being exposed at high rates via television and digital marketing. Regulations aimed at curbing exposure through these media channels are needed, as are counter advertising and prevention campaigns.

## 1. Introduction

The electronic cigarette (e-cigarette) product landscape has changed dramatically over the last decade with the introduction of more advanced devices, as well as increased availability and marketing to consumers. Use varies by age group with approximately 8.9% of U.S. young adults (ages 18–24) and 5.0% of adults (ages 25 and older) reporting the current use of e-cigarettes (every day or some days) [1]. Amongst adolescents, e-cigarettes have become the most commonly used tobacco product among high school and middle school students [2,3], with use rising 78% in one year from 11.7% in 2017 to 20.8% in 2018 [4]. Adolescents and young adults are using e-cigarettes at increasing rates, and they are more likely to experiment with e-cigarettes compared to adults [5]. E-cigarette use is associated with exposure to advertising [6,7,8,9], which individuals often report seeing or hearing about through media channels, including television, radio, print advertisements, digital marketing, and the retail environment [10,11,12].

Lenient e-cigarette advertising regulations in the U.S. have allowed e-cigarette marketing to reach its target markets, such as adult smokers who could benefit from the product use, as well as vulnerable populations, including youth and non-tobacco users. For example, an International Tobacco Control Policy Evaluation Project (ITC) (four country) study of adults found that participants from the U.S., where e-cigarette advertising was more permissive, were more likely to report exposure to e-cigarette advertising compared to participants from Canada and Australia, where e-cigarette advertising was more restrictive [13]. Most (80–88%) adolescents and college-age young adults have reported seeing at least one advertisement for e-cigarettes, most often in retail establishments, such as convenience stores and gas stations [5]. Between 2014–2016, exposure among middle and high school students was highest among retailers, the internet, and television [10]. In fact, adolescent exposure to television advertisements for e-cigarettes increased 256% between 2011 and 2013, and 321% for young adults [14]. In addition, e-cigarettes are heavily marketed online and through social media channels that are used heavily by adolescents and young adults [15,16].

The increases in reported exposure mirror the increases in e-cigarette marketing expenditures, which were minimal through 2010, but experienced a rapid increase, reaching over $1.3 billion in 2017 [15]. While print ads made up the majority of the spending, substantial expenditures were focused on television ads, an avenue that is banned for combustible cigarettes and smokeless tobacco products [5]. Additionally, the overall expenditure estimate was likely underreported, as it did not include marketing efforts aimed at online promotion, which have significantly contributed to the popularity of new e-cigarette products, such as JUUL [17].

Several studies have assessed the exposure to e-cigarette advertising across marketing channels [9,14,18,19,20,21,22,23] and its association with receptivity, curiosity, attitudes, and use, as well as smoking cessation. These studies have typically focused on a single population or age group, often with mixed results. For example, Dave and colleagues found that exposure to television e-cigarette advertisements was associated with cigarette smoking cessation amongst adults [24]. However, other studies have shown that e-cigarette advertising exposure is associated with increased urges to smoke cigarettes among cigarette smokers [22], as well as the use of other tobacco products including cigarettes, waterpipe tobacco, cigars, and polytobacco [25]. Youth-focused studies have found similar results, indicating that exposure to e-cigarette advertising is associated with the increased likelihood of subsequent e-cigarette use [25,26,27]. Despite these findings, no studies have assessed the differences in advertising exposure across age groups and media channels. We sought to fill this gap by using two nationally representative samples to: (1) examine the prevalence of e-cigarette advertising exposure among adolescents (13–17), young adults (18–25), and adults (26+) across various advertising channels; and (2) assess the associations between demographic characteristics and e-cigarette advertising exposure.

## 2. Methods and Materials

Data were collected using two national phone surveys conducted by the Carolina Survey Research Laboratory (CSRL) at the University of North Carolina at Chapel Hill, that is, one of adults ages 18 and older and the other of adolescents ages 13–17. The Institutional Review Board at the University of North Carolina at Chapel Hill approved the study.

Between September 2014 and July 2015, a national probability sample of U.S. adults was recruited using two independent and non-overlapping random-digit-dialing frames (landline and cell phone) covering approximately 98% of all U.S. households, with oversampling of regions with historically higher rates of tobacco use and poverty. Consent was obtained for adult participants. A total of 5014 adults completed the survey, of which 809 were young adults (ages 18–25) and 4205 were aged 26 and older. More information on the adult phone survey methods can be found in the literature [28,29].

A separate national probability sample of U.S. adolescents, ages 13–17, was recruited between November 2014 and June 2015. To draw the sample, we used the landline and cell phone sampling frames, as well as a targeted list of households with youth, resulting in a 98% coverage of all U.S. households. Counties with historically higher rates of tobacco use and poverty were oversampled. Verbal consent was obtained from the parent or guardian, and verbal assent was obtained from all the adolescent participants. A total of 1125 adolescents completed the survey. More information about the adolescent survey methods can be found in Jeong, 2017 [30]. We created a combined dataset consisting of data from these two surveys, which was used for this analysis, to assess the differences between adolescents, young adults, and adults.

### 2.1. Measures

The same measures were used in both the adult and adolescent telephone surveys, except where indicated below.

### 2.2. Tobacco Use

To assess e-cigarette use status, participants were read an introductory statement defining e-cigarettes that included popular e-cigarette brands. Participants who reported ever using e-cigarettes were then asked how many of the past 30 days they had used an e-cigarette device. Those who reported using an e-cigarette device in one or more days over the past 30 were considered past 30-day e-cigarette users.

All adults (young adults and older adults) were considered current cigarette smokers if they reported that they had smoked at least 100 cigarettes in their lifetime and currently smoked *every day* or *some days*. For adolescents, we first asked if they had ever tried cigarette smoking using the following item: *Have you ever tried cigarette smoking, even one or two puffs?* If the participant answered yes, we assessed current cigarette use with the question: *In the past 30 days, on how many days did you smoke cigarettes?* Adolescents who reported any use in the past 30 days were considered current cigarette smokers.

### 2.3. Advertising Exposure

To assess advertising exposure, participants were asked, “*Have you ever seen or heard any advertisements for e-cigarettes or other vaping devices?”* Response options were Yes/No. Respondents who indicated they had seen or heard an e-cigarette advertisement were asked an open-ended question, *“Where have you seen or heard advertisements for these devices?”* Respondents could list as many locations as desired. Response options were coded into the following categories of advertising channels: television, digital marketing (i.e., email, online, social media), retail establishment (i.e., gas stations, convenience store, pharmacy, grocery store, tobacco shop, vape shop, mall kiosk, or liquor store), radio, print, or billboard.

### 2.4. Demographics

We assessed the participants sex, race, ethnicity, age, and education. We used education as a marker of socioeconomic status, and for older adults and young adults we assessed their highest level of completed education. For adolescents, we assessed the highest level of maternal education. Census region was collected based on the participant’s state, and coded as Northeast, Midwest, South, or West. Sexual orientation was measured for all adults with the following item: *“The next question is about your sexual orientation. Do you consider yourself to be… Straight or heterosexual, Gay or lesbian, or Bisexual?”* Sexual attraction was measured in the adolescent sample using the following item: “*People are different in their sexual attraction to other people. Which best describes your feelings? Are you: Only attracted to females, Mostly attracted to females, Equally attracted to females and males, Mostly attracted to males, Only attracted to males, or Not sure?”* Sexual attraction was coded based on the respondents’ sex.

### 2.5. Statistical Analyses.

All analyses were conducted using SAS version 9.4 (SAS Institute, Cary, NC, USA). A standard three-step sample weighting procedure, described elsewhere, was followed to produce sampling weights [28,29]. We calculated the descriptive statistics to characterize the study sample and their exposure to e-cigarette advertising. Predictors of advertising exposure were assessed using logistic regression. Sample characteristics were summarized using unweighted proportions within each age group. Exposure to a number of advertising channels and to specific channels was summarized by a weighted analysis using PROC SURVEYFREQ. Tests for differences between age groups were performed using PROC SURVEYREG and PROC SURVEYLOGISTIC. To identify the predictors of advertising exposure, a backwards step-wise selection was done using multivariate logistic regression analyses, including the main effects of and interactions with age group for sex, race, ethnicity, education, sexual orientation, census region, e-cigarette use, and cigarette smoking. Predictors with *p*-values > 0.05 were removed sequentially, starting with the largest p-value until the model included only the effects with *p* < 0.05 or the main effects involved in interaction terms with *p* < 0.05.

### 2.6. Ethical Approval

This study was approved by the Institutional Review Board at the University of North Carolina at Chapel Hill (13-2779).

## 3. Results

The sample consisted of 1124 adolescents (ages 13–17), 809 young adults (ages 18–25), and 4186 older adults (ages 26 and older; see Table 1). Approximately half of the sample for each age group was male. Amongst adolescents, 5.2% reported past month e-cigarette use and 3.7% reported current cigarette smoking. Among young adults, 20.3% reported past month e-cigarette use and 21.7% reported current cigarette smoking. Among older adults, 8.8% reported past month e-cigarette use and 23.3% reported current cigarette smoking. Reported e-cigarette advertisement exposure was high across all three age groups, with 73.8% for adolescents, 78.2% for young adults, and 78.8% for older adults.

As shown in Table 1, television was the most frequently reported source of advertising exposure amongst all age groups (adolescents: 74.9%; young adults: 70.7%; older adults: 66.9%). Other channels of exposure included retail establishments (adolescents: 25.7%; young adults: 31.2%; older adults: 28.6%), digital marketing (adolescents: 15.6%; young adults: 18.5%; older adults: 8.9%), radio (adolescents: 14.0%; young adults: 18.3%; older adults: 16.4%), print media (i.e., magazines and newspapers); (adolescents: 12.2%; young adults: 18.6%; older adults: 27.3%), and billboards (adolescents: 11.2%; young adults: 11.6%; older adults: 11.7%).

We assessed the differences in exposure through specific advertising channels by age group (Table 2). Logistic regression models showed that adolescents were more likely than older adults to report exposure to advertisements on television (AOR: 1.62, 95% CI: 1.34–1.96) and digital marketing (AOR: 1.54, 95% CI: 1.21–1.96), but were less likely to report exposure in the retail environment (AOR: 0.77, 95% CI: 0.64–0.93), radio (AOR: 0.76, 95% CI: 0.60–0.96), and print media (AOR: 0.37, 95% CI: 0.29–0.47). Compared to young adults, adolescents were less likely to report exposure in the retail environment (AOR: 0.63, 95% CI: 0.49–0.80) and print media (AOR: 0.62, 95% CI: 0.45–0.86). Young adults were more likely than older adults to report exposure on television (AOR: 1.30, 95% CI: 1.06–1.60) and digital marketing (AOR: 1.78, 95% CI: 1.38–2.30), and were less likely to report exposure via print media (AOR: 0.59, 95% CI: 0.46–0.76). There were no differences between the age groups in exposure to e-cigarette advertising on billboards.

### Predictors of E-cigarette Advertising Exposure.

Table 3 presents the odds of e-cigarette advertising exposure by participant characteristic.

The final model included age, sex, race, education, and two interactions (age by sex and age by cigarette smoking status). Each of the covariates had a significant main effect on the exposure to e-cigarette advertising. Age was significantly associated with exposure among all age groups, with older adults having the highest odds of exposure (AOR: 4.09; 95% CI: 3.60–4.65), followed by young adults (AOR: 3.56, 95% CI: 2.79–4.55), and then adolescents (AOR: 1.90 95% CI: 1.31–2.76). Sex was significantly associated with exposure among males (AOR: 3.41; 95% CI: 2.84–4.10) and females (AOR: 2.68, 95% CI: 2.23–3.23). Race was significantly associated, with Whites having the highest odds of exposure (AOR: 3.77; 95% CI: 3.22–4.41), followed by Blacks (AOR: 3.36, 95% CI: 2.72–4.16), and then other races (AOR: 2.19, 95% CI: 1.72–2.79). Education was significantly associated with exposure amongst participants, with the some college characteristic having the highest odds of exposure (AOR: 3.62; 95% CI: 2.96–4.42), followed by participants with a 4 year college degree or more (AOR: 3.25, 95% CI: 2.66-3.95), and then participants with a high school diploma or less (AOR: 2.36, 95% CI: 1.97–2.83). Smoking status was also significant amongst current smokers (AOR: 3.09; 95% CI: 2.31–4.15) and non-smokers (AOR: 2.96, 95% CI: 2.65–3.30). Each of the interactions were significantly associated with e-cigarette advertising exposure, except for adolescents who were current smokers.

**Figure 1:**
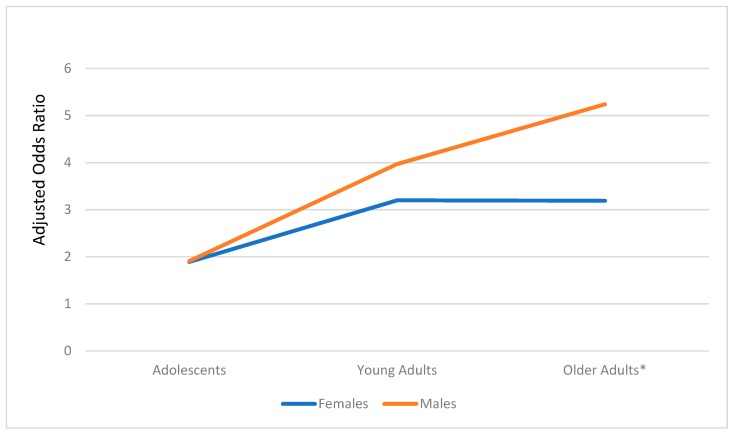
Exposure to e-cigarette advertising: Age group by sex interaction. *: *p* < 0.001.

**Figure 2:**
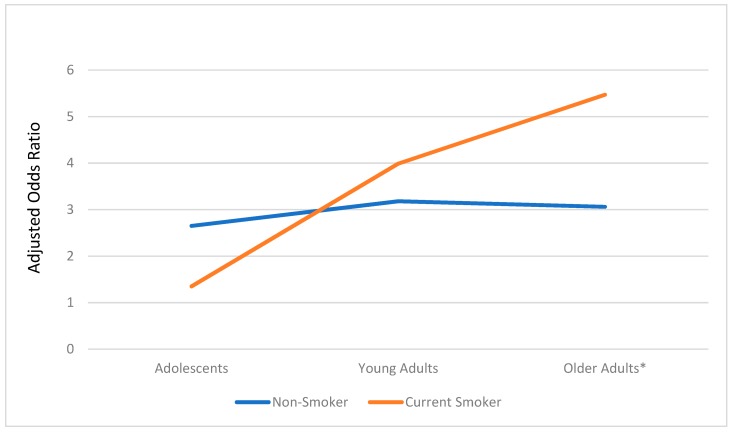
Exposure to e-cigarette advertising: Age group by cigarette smoking status interaction. *: *p* < 0.001.

Table 4 presents the results of the model testing differences amongst the interactions. The interaction between age and sex was only significant in the older adults, where males had more exposure than females (Figure 1). Similarly, as shown in Figure 2, the interaction between age and cigarette smoking status was only significant among older adults, where older adult smokers had increased odds of exposure compared to older adult non-smokers.

## 4. Discussion

Exposure to e-cigarette advertising, at least once, is pervasive among adolescents, young adults, and older adults with estimates ranging from 73.8–78.8%, similar to other studies of adolescents and young adults [5,10]. In our study, the odds of exposure was significant among all age groups, gender, races, educational attainment, and smoking status. We also found differences in the advertising channels through which these groups reported seeing or hearing e-cigarette advertisements. Television was the largest source of advertisement exposure for all the age groups, while retail establishments were the second most common source. These findings are similar to other studies [5,10,27]. With more e-cigarette companies launching television campaigns, most recently for JUUL and Vuse Alto to reach adult smokers, research is needed to document viewership and campaign content to ensure adolescents, young adults, and non-tobacco users are not targeted [31].

In addition to television exposure, approximately 25–30% of participants reported exposure at retail establishments. Many e-cigarette brands are available and advertised in traditional tobacco retailers, as well as through specialty vape shops. Documenting the types of advertising being made in these venues is important because the messages may differ by media channel. E-cigarettes have been marketed online and in print media as cessation aids, smoke-free, and healthier than combustible cigarettes [32,33]. However, little is known about the e-cigarette claims made on television and the retail environment. More research is needed to determine if the claims are similar across channels, which could aid the Food and Drug Administration (FDA) in developing new regulations and enforcing existing ones.

Finally, e-cigarette brands are reaching consumers online, which our study shows means reaching a younger audience, including adolescents and young adults. Although online advertising is difficult to regulate, in recent months the FDA has identified several e-cigarette companies, including JUUL Labs, Altria, and Japan Tobacco International to “revise current marketing practices to help prevent use by minors,” inclusive of eliminating online sales and modifying marketing practices that target adolescents and young adults [34,35,36]. Although this reactive action may help to prevent future exposure, it cannot negate the exposure that millions of youth have already experienced.

E-cigarette advertising exposure amongst adolescents and nicotine naïve individuals is concerning, no matter which advertising channel is used for delivery. This study provides valuable information as to who is most likely to be exposed to e-cigarette advertising through various advertising channels, helping to identify specific channels that could be targets for regulation. The study also identifies opportunities for targeted marketing approaches for counter-advertising on product risks to susceptible audiences. For example, we found that adolescents were more likely than adults to report exposure to advertising on television and digital marketing, but less likely to report exposure in the retail environment, radio, and print media. This suggests that regulations aimed at reducing the digital marketing and television content promoting e-cigarettes may be one strategy to reduce adolescent advertising exposure. Some progress has been made in this area with popular e-cigarette brands, such as JUUL, halting social media marketing aimed at adolescents and young adults [37]. However, these restrictions are brand-specific and voluntary. What is needed are regulations aimed at the entire e-cigarette category to ensure that e-cigarette advertising and promotions are not targeting adolescents and young adults via the media channels that they often use.

Our findings suggest that some channels (e.g., television and digital marketing) could be effective outlets for e-cigarette counter-advertising that is targeting adolescents. While this approach may increase the opportunity to reach the intended audience, any e-cigarette counter-advertising released via mainstream media could be viewed by unintended audiences, such as exclusive, established smokers. Thus, it is important to measure the exposure of the targeted audience, as well as unintended audiences, to determine the effect on these populations. The FDA is currently using such an approach, sponsoring counter-advertising on television and online through the Real Cost campaign, which highlights the dangers of e-cigarette use, explicitly aimed at adolescents who are nicotine naïve. In implementing this approach, the FDA has implemented targeted marketing in an effort to ensure specific audiences are exposed to the campaign, whilst unintended audiences are not. Further research is needed to determine if this approach focused on message dissemination via specific channels is effective.

Finally, we found that e-cigarette marketing is also reaching the intended audiences of adult smokers. We found a strong age by cigarette smoking interaction, with older adult smokers having a higher odds of exposure compared to older non-smokers, which is an important finding if these individuals are convinced to switch to e-cigarettes. While the e-cigarette advertisements could be related to behavioral outcomes, such as interest in trying e-cigarettes, they could also serve as cues to smoke. More research is needed to determine how e-cigarette advertisement exposures are affecting smokers.

## 5. Conclusions

Although e-cigarette advertising appears to be reaching the intended audience of adult smokers, vulnerable populations are being exposed at high rates via television and digital marketing. Regulations that are aimed at curbing exposure through these media channels are needed, as are counter advertising and prevention campaigns.

### Limitations

Our findings should be interpreted in light of the study limitations. Since our study was part of a larger parent study focused on all tobacco products, we asked limited questions regarding exposure to e-cigarette advertising to limit participant burden. We assessed lifetime exposure to e-cigarette advertising instead of a more recent time point, such as past 30 day or past 6 months, which would have provided more temporal estimates of exposure. Additionally, we assessed e-cigarette advertisement exposure with an open-ended question instead of fixed response options, which may have introduced recall bias that could result in conservative estimates of exposure. Since we surveyed adolescents and adults, we used a slightly different methodology to measure some constructs for adolescents and adults (i.e., current smoking status), which may have introduced some variation. This survey was cross-sectional, thereby limiting our ability to infer causality [38,39]. Finally, the data were collected from 2014–2015 and may not necessarily reflect the current patterns of advertising and use rates.

## Figures and Tables

**Table 1:** Sample characteristics (unweighted).

Characteristic	Adolescents (13–17) (*N* = 1124)	Young Adults (18–25) (*N* = 809)	Older Adults (26+) (*N* = 4186)
Overall	18.4%	13.2%	68.4%
Age	15.1 years	21.4 years	50.6 years
Sex			
Male	49.9%	50.3%	46.8%
Race			
White	80.1%	60.8%	71.2%
African American	10.6%	23.7%	18.8%
Other	9.3%	15.5%	10.0%
Ethnicity			
Hispanic	7.6%	12.5%	7.9%
Education			
High school or less	23.0%	43.4%	33.6%
Some college	19.6%	40.4%	28.8%
4-year college or more	57.4%	16.2%	37.7%
U.S. Region			
Northeast	13.7%	10.4%	10.7%
Northwest	25.0%	19.5%	19.4%
South	48.9%	54.6%	53.4%
West	12.4%	15.5%	16.5%
Sexual Orientation/Attraction			
Heterosexual	95.2%	92.7%	96.9%
Past 30-day e-cigarette user	5.2%	20.3%	8.8%
Current cigarette smoker	3.7%	21.7%	23.3%
Exposure to advertising channel	73.8%	78.2%	78.8%
Television	74.9%	70.7%	66.9%
Retail establishments	25.7%	31.2%	28.6%
Digital marketing	15.6%	18.5%	8.9%
Radio	14.0%	18.3%	16.4%
Print media	12.2%	18.6%	27.3%
Billboard	11.2%	11.6%	11.7%

**Table 2:** Adjusted odds ratios (AOR) for advertising channel exposure by age group.

Source	Adolescent vs Young Adult AOR (95% CI) *p*-Value	Adolescent vs Older Adult AOR (95% CI) *p*-Value	Young Adult vs Older Adult AOR (95% CI) *p*-Value
Television	1.24 (0.96,1.61) 0.10	1.62 (1.34,1.96) < 0.01	1.30 (1.06, 1.60) 0.01
Retail establishments	0.63 (0.49, 0.80) < 0.01	0.77 (0.64, 0.93) 0.01	1.17 (1.01-1.49) 0.04
Digital marketing	0.87 (0.64, 1.17) 0.36	1.54 (1.21, 1.96) < 0.01	1.78 (1.38, 2.30) < 0.01
Radio	0.78 (0.57, 1.07) 0.12	0.76 (0.60, 0.96) 0.02	0.97 (0.76,1.24) 0.79
Print media	0.62 (0.45,0.86) < 0.01	0.37 (0.29,0.47) < 0.01	0.59 (0.46,0.76) < 0.01
Billboard	0.97 (0.68,1.38) 0.86	0.96 (0.74,1.24) 0.75	0.99 (0.74,1.33) 0.95

**Table 3:** Estimated odds of exposure to e-cigarette advertising by participant characteristic.

Participant Characteristic	AOR (95% CI)	*p*-Value *
**Age**		
Adolescents (13–17 years old)	1.90 (1.31, 2.76)	0.0007
Young Adults (18–25 years old)	3.56 (2.79, 4.55)	<0.0001
Older Adults (26+ years old)	4.09 (3.60, 4.65)	<0.0001
**Sex**		
Female	2.68 (2.23, 3.23)	<0.0001
Male	3.41 (2.84, 4.10)	<0.0001
**Race**		
White	3.77 (3.22, 4.41)	<0.0001
African American	3.36 (2.72, 4.16)	<0.0001
Other	2.19 (1.72, 2.79)	<0.0001
**Education**		
High school or less	2.36 (1.97, 2.83)	<0.0001
Some college	3.62 (2.96, 4.42)	<0.0001
4-year college or more	3.25 (2.66, 3.95)	<0.0001
**Cigarette smoking status**		
Current cigarette smoker	2.96 (2.65, 3.30)	<0.0001
Non-smoker	3.09 (2.31, 4.15)	<0.0001
**Age by sex interaction**		
Adolescent x female	1.89 (1.26, 2.83)	00.0021
Adolescent x male	1.91 (1.27, 2.88)	00.0020
Young Adult x female	3.20 (2.32, 4.42)	<0.0001
Young adult x male	3.97 (2.95, 5.33)	<0.0001
Older adult x female	3.19 (2.78, 3.67)	<0.0001
Older adult x male	5.24 (4.43, 6.21)	<0.0001
**Age by cigarette smoking status interaction**		
Adolescent x non-current smoker	2.65 (2.20, 3.20)	<0.0001
Adolescent x current smoker	1.36 (0.66, 2.78)	0.4010
Young Adult x non-current smoker	3.18 (2.57, 3.94)	<0.0001
Young adult x current smoker	3.99 (2.58, 6.17)	<0.0001
Older adult x non-current smoker	3.06 (2.74, 3.42)	<0.0001
Older adult x current smoker	5.47 (4.42, 6.77)	<0.0001

* *p*-values test hypothesis that odds of exposure equal 1.

**Table 4:** Estimated odds of exposure in each age group by sex and cigarette smoking status.

Sex	Adolescent AOR (95% CI)	Young Adult AOR (95% CI)	Older Adult AOR (95% CI)
Male	1.91 (1.27, 2.88)	3.97 (2.95, 5.33)	5.24 (4.43, 6.21)
Female	1.89 (1.26, 2.83)	3.20 (2.32, 4.42)	3.19 (2.78, 3.67)
*p*-value *	0.95	0.26	<0.0001
**Cigarette Smoking Status**			
Non-Smoker	2.65 (2.20, 3.20)	3.18 (2.57, 3.94)	3.06 (2.74, 3.42)
Current Smoker	1.36 (0.66, 2.78)	3.99 (2.58, 6.17)	5.47 (4.42, 6.77)
*p*-value *	0.07	0.36	<0.0001

* *p*-values test the difference between odds within sex and within cigarette smoking status.
